# Fibulin-2 is required for basement membrane integrity of mammary epithelium

**DOI:** 10.1038/s41598-018-32507-x

**Published:** 2018-09-20

**Authors:** Ayman M. Ibrahim, Salwa Sabet, Akmal A. El-Ghor, Nora Kamel, Shady E. Anis, Joanna S. Morris, Torsten Stein

**Affiliations:** 1 Zoology Department, Faculty of Science, Cairo University, Giza, 12613 Egypt; 2 Institute of Cancer Sciences, College of MVLS, University of Glasgow, Glasgow, G12 8QQ UK; 3 Department of Pathology, National Research Center, Cairo, 12622 Egypt; 4 Department of Pathology, Faculty of Medicine, Cairo University, Cairo, 11562 Egypt; 5 School of Veterinary Medicine, University of Glasgow, Bearsden Road, Glasgow, G61 1QH UK

## Abstract

Fibulin-2 (FBLN2) is a secreted extracellular matrix glycoprotein which has been associated with tissue development and remodelling. In the mouse mammary gland, FBLN2 can be detected during ductal morphogenesis in cap cells and myoepithelial cells at puberty and early pregnancy, respectively. In an attempt to assign its function, we knocked down Fbln2 in the mouse mammary epithelial cell line EpH4. FBLN2 reduction led to an increase in the size of spheroidal structures when compared to scrambled control shRNA-transduced cells plated on Matrigel matrix. This phenotype was associated with a disruption of the collagen IV sheath around the epithelial spheroids and downregulation of integrin β1, suggesting a role for FBLN2 in stabilizing the basement membrane (BM). In contrast to mice, in normal adult human breast tissue, FBLN2 was detected in ductal stroma, and in the interlobular stroma, but was not detectable within the lobular regions. In tissue sections of 65 breast cancers FBLN2 staining was lost around malignant cells with retained staining in the neighbouring histologically normal tissue margins. These results are consistent with a role of FBLN2 in mammary epithelial BM stability, and that its down-regulation in breast cancer is associated with loss of the BM and early invasion.

## Introduction

Breast cancer is one of the most widespread types of cancer in females worldwide and one of the leading causes of cancer-associated deaths^1,2^. The tumour microenvironment (TME) is an important contributor to breast cancer formation and progression involving multiple cell types, as well as growth factors and modulators of the extracellular matrix (ECM)^3,4^. ECM proteins themselves also play a central role in the TME. For instance, periostin (POSTN), fibronectin (FN), tenascin-c (TN-C), and hyaluronan are all well documented components of the metastatic niche in cancerous tissues such as breast cancer^5,6^. However, our understanding of the contribution that the individual ECM components make to disease development and progression is still limited.

Fibulin-2 (FBLN2) is a secreted extracellular glycoprotein originally identified in the embryonic endocardial cushion tissue and the heart valves of adult mice and humans^7^. FBLN2 has been associated with the development and remodelling of tissues, as it is expressed at sites of epithelial-mesenchymal transition during endocardium formation in the developing heart and during neural crest development^8^. It is also expressed by the smooth muscle precursor cells of developing aortic arch vessels^9^.

In the mouse mammary gland, FBLN2 has been specifically detected in and around the cap cells of the terminal end buds during puberty in regions where the basement membrane (BM) is formed along the newly developing mammary ductal epithelium, as well as in myoepithelial cells during early pregnancy when the ductal ECM is remodelled to enable lateral branching to occur^10^. This expression pattern indicates a possible role in morphogenesis of the newly formed ducts. As FBLN2 has been shown to bind and bridge other BM proteins, including FN, nidogens, versican, and hyaluronan^11^, and links these proteins to form stable ECM networks^12^, we hypothesised that FBLN2 may be important for the formation of a new stable BM during mammary gland morphogenesis. However, *fbln2 KO* mice have no major mammary phenotype^10^ as the loss of FBLN2 is compensated by a relocation of other fibulin proteins, in particular FBLN1^11^, while knockout of FBLN1 itself is lethal due to loss of BM in small blood vessels leading to haemorrhage^13^. Therefore, there is a need for *in vitro* assays to be able to assess the possible function of FBLN2 in mammary gland morphogenesis.

BM integrity is crucial for the suppression of tumour invasiveness, and BM breakup and loss is a major hallmark of cancer progression^14^. Little is known about FBLN2′s role in cancer, though a role in tumour suppression has been suggested by recent studies on nasopharyngeal carcinoma^15^, colorectal cancer^16^, and in breast cancer cells^17^.

In this study, we further investigated the function of FBLN2 in normal mammary epithelial cells by knocking down FBLN2 in the mouse mammary epithelial cell line EpH4, and assessed its expression in normal and cancerous human breast tissue. Here we show that reduced FBLN2 levels in normal mammary epithelial cells are associated with a significant reduction in integrin β1 (ITGβ1) and a discontinuous BM, and that FBLN2 expression is gradually lost in areas of tumour invasion. Our results are consistent with a role for FBLN2 in retaining BM integrity, and demonstrate an association between loss of FBLN2 expression and loss of BM in the progressing malignant breast tissue.

## Results

### FBLN2 knockdown induces enlarged cell morphology

**Figure 1:**
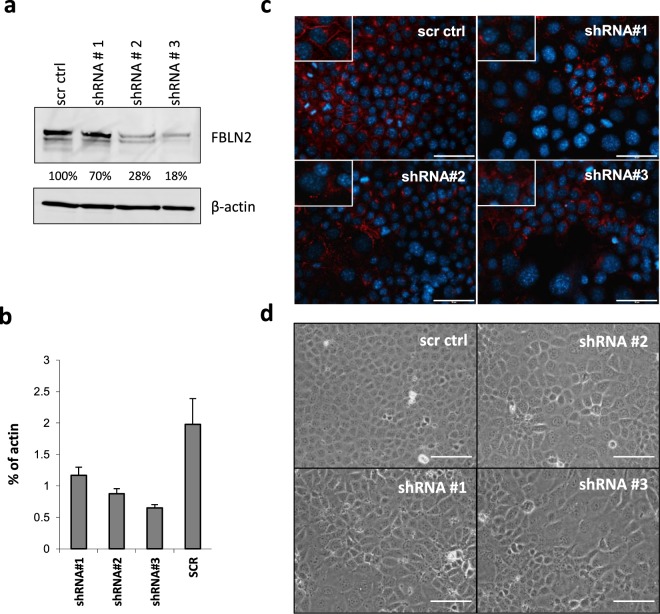
Confirmation of FBLN2 KD and the effect on cell size. (**a**) FBLN2 and β-actin expression in three separate EpH4 lines stably transduced with FBLN2 shRNAs (#1-3) or with scr control as measured by western blot. Percentages show the relative expression of FBLN2 in the stably transduced cells normalised to β-actin expression (from same gel) and as measured by ImageJ software. (**b**) Quantitative PCR analysis of *Fbln2* RNA expression in the three stably transduced lines with FBLN2 shRNAs compared to scr ctrl; expression was quantified relative to β-actin. Error bars show standard deviation for technical replicates. (**c**) Immunofluorescent analysis of FBLN2 expression in the stable KD cell lines, showing the different levels of membrane-associated FBLN2 in the three KD cell lines. (**d**) Phase contrast microscopy of cells showing the differences in morphology upon plating on plastic. Bars represent 100 μm.

Despite FBLN2′s distinct and selective expression around newly growing mammary epithelium, *fbln2* KO mice did not display any mammary phenotype^10^. To investigate a possible role for FBLN2 during mammary epithelial development, we stably transduced FBLN2-expressing mammary epithelial EpH4 cells with lentiviral shRNA constructs against *Fbln2* (or scr control) and selected for stable transduction and shRNA expression by puromycin treatment. The three constructs reduced FBLN2 protein expression by 30–80% in 2D culture, and with a corresponding reduction at RNA level (Fig. 1a,b). Down-regulation of FBLN2 protein expression was further confirmed in the stably transfected clones by a strong reduction in membrane-associated FBLN2 in immunofluorescent analysis (Fig. 1c). Microscopic analysis of the cells at confluency showed that Fbln2 KD cells were consistently larger than scr shRNA control cells and had enlarged nuclei (Fig. 1d), though surprisingly this cell size enlargement was not readily detectable by flow cytometric analysis (Supplementary Fig. 1).

### FBLN2 knockdown leads to enlarged colony formation in 3D culture

**Figure 2:**
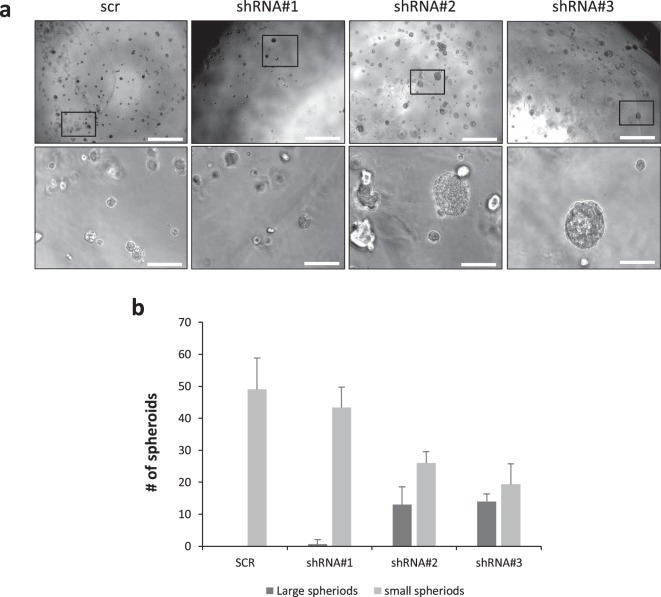
Size increase of Matrigel-embedded EpH4 cells associated with FBLN2 KD. **(a)** Phase contrast microscopy of the stably transduced EpH4 cells grown in Matrigel matrix expressing scr vector, shRNA#1, shRNA#2, or shRNA#3, showing the size increase in spheroids upon Fbln2 KD. Pictures are representative of three independent experiments. Scale bars represent 500 µm (top) and 100 µm (bottom). **(b)** Quantification of small and large spheroidal structures of stably transduced EpH4 cells grown in Matrigel matrix. Bars are standard error for three biological replicates.

FBLN2 is a secreted BM protein and any effect on cell behaviour may therefore become mostly apparent when cells are grown in a 3D environment, which allows the formation of a BM around spherical cell colonies. KD cells were therefore analysed in a 3D assay involving growth in Matrigel matrix. While scr shRNA control-transduced EpH4 cells formed small evenly shaped round spheroids, the size of the spheroids was consistently increased in the Fbln2 KD cells with the strongest suppression of FBLN2 compared to the scr shRNA control cells (Fig. 2).

### EpH4 Fbln2 KD cells when co-cultured with fibroblast show stellate branch pattern

**Figure 3:**
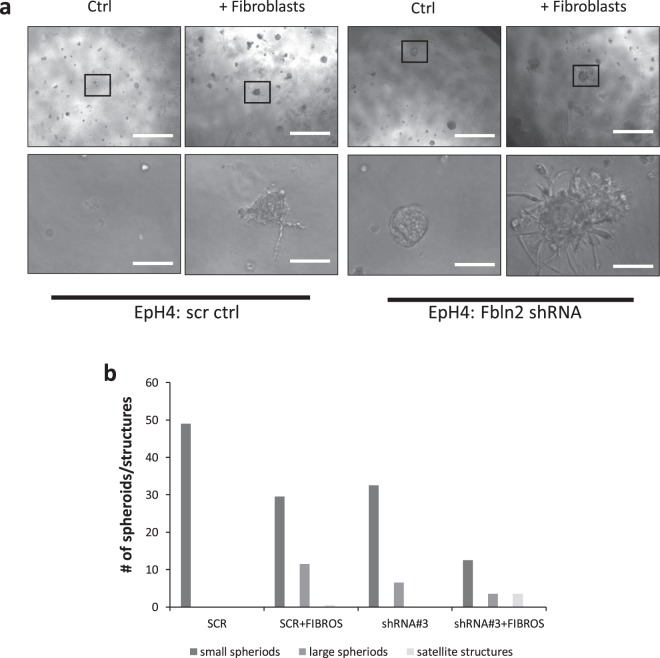
EpH4 Fbln2 KD cells when co-cultured with fibroblast show a stellate branch pattern. Phase contrast microscopy of the stably transduced EpH4 cells (scr and cells with lowest (18%) expression of FBLN2) grown on Matrigel matrix, alone and co-cultured with fibroblasts. Bars represent 500 μm (top) and 100 μm (bottom). **(b)** Quantification of small large spheroidal structures and satellite structures with or without fibroblasts in control (scr) and shRNA#3 cells. The numbers are representative of two biological replicates.

To investigate whether KD of FBLN2 affected the epithelial cells’ ability to branch, we co-cultured FBLN2 KD and scr control EpH4 cells with primary mammary fibroblasts in a Matrigel matrix. Fibroblasts had previously been shown to induce mammary epithelial branching in 3D culture when co-cultured with mammary epithelial organoids^18^. While the increased spheroidal size was still evident in Fbln2 KD cells, co-culture with fibroblasts led to a strongly increased stellate branch pattern compared to control scr ctrl cells, with either individual cells or thin tubules extending from the spheroids into the surrounding matrix (Fig. 3).

### FBLN2 knockdown affects basement membrane integrity

**Figure 4:**
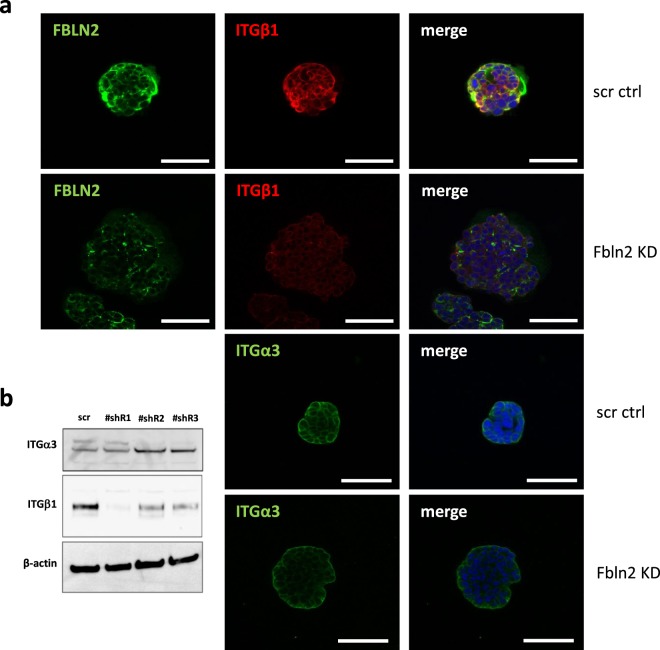
Fbln2 KD is coupled with a reduction in Integrin beta 1 expression but not Integrin alpha 3. (**a**) Immunofluorescent analysis of FBLN2, ITGβ1 and ITGα3 expression in the stable KD cell lines with the lowest (18%) FBLN2 expression. Bars represent 50 μm (**b**) Western blotting analysis of ITGα3 and ITGβ1 relative to actin in protein extracts from 2D-plated scr ctrl and Fbln2 KD cells. (Note: The ITGα3 was measured on the same membrane as FBLN2 in Fig. 1a after antibody stripping; ITGβ1 and actin are from the same blot, though from equally loaded neighbouring parts of the gel).

Fibulins can interact directly with integrins and contribute to BM integrity^9–11^, with FBLN2 KO mice showing a blistering phenotype of the epidermis of newborn mice similar to that of ITGα3 KO mice^19^. Integrin expression was therefore investigated in EpH4 spheroids. Although ITGα3 expression was increased in total protein extracts from 2D-plated FBLN2 KD cells with the strongest reduction in FBLN2 protein, this increase was not measureable when spheroids of the Matrigel-grown cells were assessed by immunofluorescence, while the strong reduction in FBLN2 expression was still evident (Fig. 4a,b). In contrast, ITGβ1 expression was strongly reduced in the FBLN2 KD cells as shown by western blot and when Matrigel-embedded structures were examined by immunofluorescence (Fig. 4a,b).

**Figure 5:**
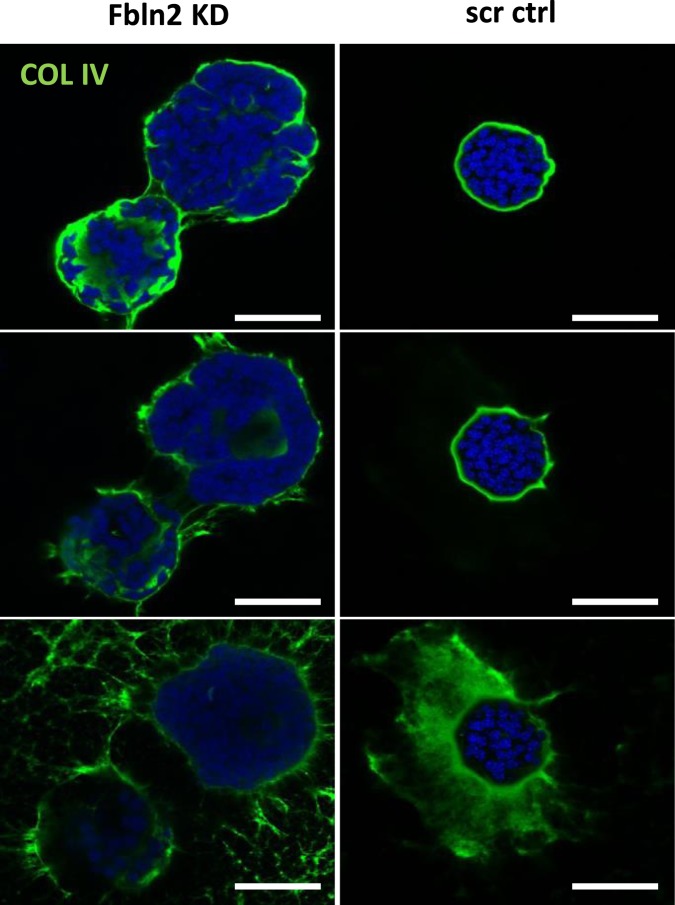
Fbln2 KD affects the integrity of COLIV sheath in Matrigel-embedded EpH4 cells. Immunofluorescent analysis of COLIV expression through three confocal optical z-sections in the stable KD cell lines with the lowest FBLN2 expression, plated in Matrigel matrix. Bars represent 50 μm.

To test whether BM integrity was also affected by the decrease in FBLN2, 3D colonies of KD and control cells were stained for the basal membrane protein COLIV, a key component of the lamina densa. While scr control cells showed an evenly formed COLIV layer around the spheroids, FBLN2 KD cells had a discontinuous and irregular COLIV layer (Fig. 5), which was independent of the colony size. This finding is consistent with a role for FBLN2 in the formation of a stable COLIV-layer and hence BM integrity.

### FBLN2 expression in human breast cancer

The BM has been recognised as an important barrier that suppresses tumour invasion and hence metastasis formation^14,20^. The role of FBLN2 in breast cancer has so far not been well studied and recent reports are contradictory about its contribution to tumour development^17,21,22^. Our *in vitro* results supported a role for FBLN2 in BM integrity, and we therefore hypothesised that in breast cancer FBLN2 expression would be expected to be reduced in line with progression from normal to invasive tissue. We therefore assessed FBLN2 expression in formalin-fixed histological sections from 65 breast cancer patients with invasive breast cancer with adjacent ductal carcinoma *in situ* (DCIS) as well as morphologically normal breast tissue to establish its role in disease progression to invasive cancer (Table 1).

**Table 1:** Distribution of FBLN2 expression in morphologically normal and cancerous breast.

Intensity Score	0	1	2	3	N/A	Total
*morph*. *normal ducts*	2	0	20	40	3	65
*DCIS*	42	14	0	0	9	65
*invasive*	45	16	0	0	4	65

FBLN2 staining intensity in morphologically normal margins, areas of DCIS and in invasive areas of 65 breast cancer patients’ tissue sections (0 = negative; 1 = weak; 2 = intermediate; 3 = strong). NA: No available data.

**Figure 6:**
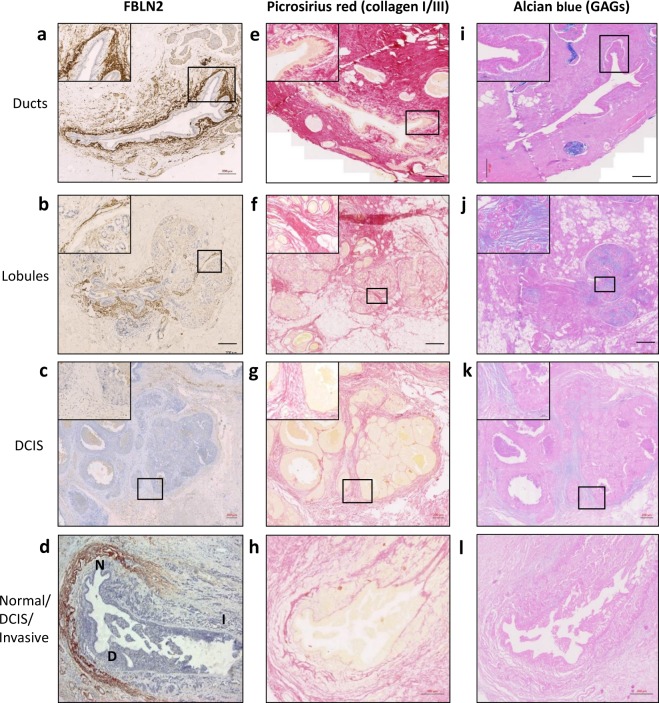
Immunohistochemical assessment of FBLN2 expression in relation to the collagen fibre network in normal ducts and lobules. (**a**–**d**) Immunohistochemical analysis of FBLN2 in normal ducts and lobules in regions of ducal carcinoma *in situ* (DCIS) and the boundary between a duct and the invasive area. (**e**–**h**) Picrosirus staining (red) showed a coherent fibrous collagen network in normal ducts and interlobular stroma where FBLN2 is expressed, but a much less coherent network in intralobular stroma, around DCIS and in invasive regions. (**i**–**l**) Alcian blue staining showed significant amount of GAGs (blue) in the stroma surrounding the tumour tissue where FBLN2 was absent and the collagen network was less intact. ‘N’ (normal), ‘D’ (DCIS) and ‘I’ (invasive) describe the different areas within a section. Bars represent 200 μm.

Immunohistochemical examination of morphologically normal breast tissue showed strong staining for FBLN2 around normal ducts at the myoepithelial cell/BM interface, as shown by staining of the same tissues for smooth muscle actin (SMA) and vimentin (Vim), (Fig. 6 and Supplementary Fig. 2) as well as in the interlobular stroma, where the ECM is intact and the collagen I/III network is unimpaired/coherent as demonstrated by the intensity of fibrous collagen staining with picrosirus red^23^. In contrast, FBLN2 expression was decreased in the intralobular stroma where the collagen I/III network was much less coherent (Fig. 6a,b,e,f). In DCIS and invasive tumour regions FBLN2 expression was either undetectable or weak when compared to the expression around normal ducts (Table 1). This was particularly obvious at the interface between normal ducts and tumour tissue, where ductal expression of FBLN2 was lost in regions of DCIS/invasive carcinoma (Fig. 6c,d). Pearson’s test showed a negative significant correlation (P < 0.001) between the level of FBLN2 expression and tumour grade (Supplementary Table 2).

To verify that the absence of FBLN2 in invasive cancerous areas was not a technical artefact, we stained 10 cancer tissue sections for other known markers of invasive and metastatic spread. Glycosaminoglycans (GAGs) are known modulators of cell-ECM interaction associated with cancerous tissues^24^, and we therefore assessed GAGs presence in relation to FBLN2 expression by Alcian blue staining. Morphologically normal tissue sections showed little GAGs staining in areas where FBLN2 staining was strong (Fig. 6I,j). In contrast, significant levels of GAGs were detected in the stroma surrounding tumour tissue within all tested sections with the less intact network of fibrous collagen, and where FBLN2 expression was absent (Fig. 6g,k,h,l). At the same time, the BM-associated ECM proteins tenascin C (TN-C) and periostin (POSTN) expression, whose expression has been associated with metastatic spread^25,26^, were all strongly expressed in these regions (Supplementary Figs 3 and 4).

### FBLN2 localizes to collagen IV/BM in human mammary tissue

**Figure 7:**
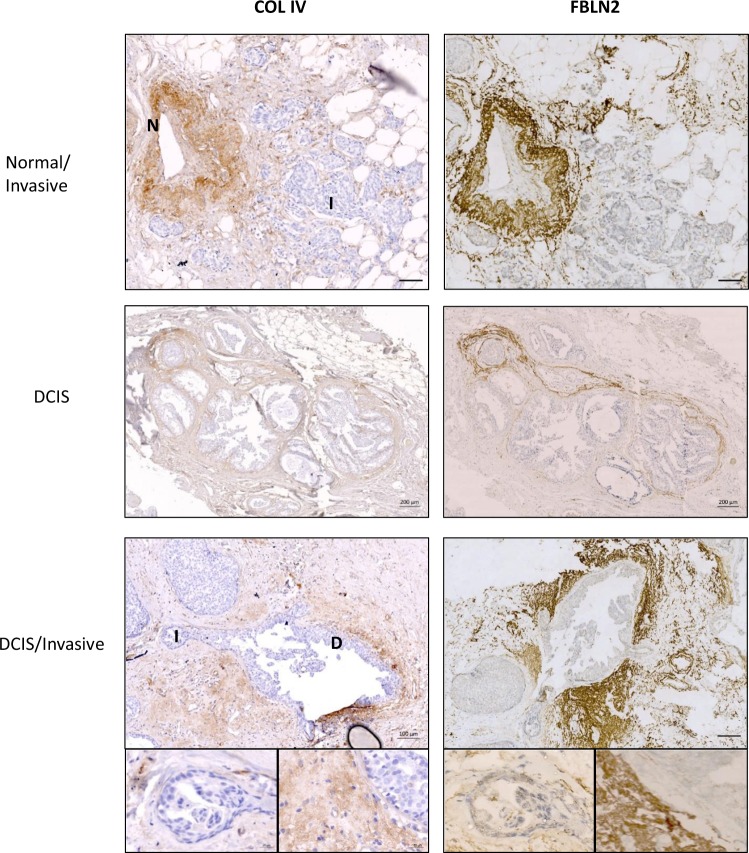
Loss of FBLN2 and COLIV expression are associated with BM break up. Immunohistochemical analysis of FBLN2 and COLIV in breast cancer regions (DCIS) and morphologically normal tissue at three representative regions. ‘N’ (normal), ‘D’ (DCIS) and ‘I’ (invasive) describe the different areas within a section. Bars represent 100 μm and 200 μm.

Our *in vitro* data on EpH4 cells suggested that FBLN2 was necessary for a stable BM. To investigate whether FBLN2 expression was also associated with an intact BM in the human breast, we assessed co-expression of FBLN2 and COLIV in 10 out of the 65 human breast cancer tissues and histologically normal tissue margins, which were randomly chosen. Consistent with a role for FBLN2 in BM stability, both proteins co-localised at and near the BM of morphologically normal ducts, while loss of FBLN2 staining was evident in areas of invasion, where breakup of the normal ductal structure was evident by the loss of COLIV staining, with complete loss of both proteins in areas of invasion (Fig. 7).

### High levels of *Fbln2* mRNA are associated with better distant metastasis-free survival in lymph node negative and intermediate grade breast cancer patients

To further test our hypothesis of FBLN2′s positive contribution to controlling cancer growth and progression we assessed whether high *Fbln2* mRNA expression correlated with improved distant-metastasis-free survival (DMFS) in patients with breast cancer of different grades and LN status, using the Kaplan Meier plotter dataset^27^ (Supplementary Fig. 5). High levels of *Fbln2* mRNA expression were indeed significantly associated with improved DMFS in patients with negative LN status (*P* = 0.03; n = 988), as well as in patients with intermediate grade (Grade II, *P* = 0.05; n = 546), while there was a statistically insignificant trend towards better survival in low grade breast cancer patients (Grade I, *P* = 0.14; n = 188). In contrast, in the high grade breast cancer patient group (Grade III (*P* = 0.023; n = 458)) higher *Fbln2* mRNA levels were significantly associated with a poorer outcome, and showed a statistically insignificant trend with poorer DMFS in patients with positive LN status (*P* = 0.14; n = 382). Although not fully conclusive, this indicates that FBLN2 may play different roles at the early and advanced stage of breast cancer progression.

## Discussion

We have previously shown that FBLN2 is upregulated in terminal end buds compared to ductal epithelium at puberty, as well as in myoepithelial cells of early pregnancy ducts when the first morphological changes become microscopically visible^10^, suggesting a role in the formation of the new BM during ductal (branching) morphogenesis. However, *fbln2* knockout mice did not show any morphological defects likely due to compensation effects by other fibulins including FBLN1^10^. Therefore, despite FBLN2’s highly distinct expression and localisation in the mouse mammary gland, FBLN2′s role in mammary tissue morphogenesis and homoeostasis has not been established. In this study, we investigated the effect of Fbln2 KD in mouse mammary epithelial cells and further studied its expression in human breast cancer.

EpH4 cells were the only normal mouse mammary cell line in our laboratory that expressed detectable levels of FBLN2 on mRNA and protein level (data not shown). We therefore used this cell line to produce stable knockdown cells to study FBLN2′s possible role in the mammary gland. Reduced expression of FBLN2 in EpH4 cells grown in Matrigel produced larger spheroids than cells transfected with scrambled control shRNA. Although this could be attributed to a higher growth rate, no difference between control and KD EpH4 cells was identified, when the proliferation rate of each line was assessed (data not shown). In 2D culture, cells were larger and had enlarged nuclei although this was not observed when analysed by flow cytometry, indicating a larger cell spread. The change in morphology might be due to altered adherence to the plastic upon Fbln2 KD, possibly via changes in keratin expression and the cell cytoskeleton^28,29^.

On Matrigel, the increase in size of spheroids was associated with disruption of the surrounding COLIV sheath. This was accompanied by a significant reduction in integrin β1 expression. This increase in size was an unexpected result as block of integrin β1 binding to the ECM has previously been shown to result in suppressed growth^30^. However, the disruption of the BM after reduction in ITGβ1 is consistent with previous reports of mice lacking ITGα3/β1, which showed that reduced FBLN2 expression was associated with loss of epidermal BM integrity^19^. In contrast though, integrin α3 was still expressed in our KD cells when grown in an organotypic model and appeared up-regulated in cells grown on plastic. Whether other β-type integrins were upregulated to compensate for reduced levels of integrin β1 is unknown.

Although a direct interaction between fibulins and COLIV in BM has not been established, there is evidence for an indirect interaction. COLIV interacts with and binds to other BM components such as nidogen, perlican and laminin^11,31^, all of which are known binding partners of FBLN2. E.g. Nidogen is required for the link between COLIV and laminin for proper epithelial branching upon BM assembly^32^. Our results are consistent with this link being important for BM integrity, and that its loss can have a drastic effect on the growth of epithelial cells.

Moreover, cells interact with the BM through integrins^33,34^. As ITGβ1/α3 is a receptor for laminin, collagen and FN^35,36^, down regulation of ITGβ1 in Fbln2 KD cells could potentially affect the integrity of COLIV around epithelial cells disrupting the BM and impairing the interaction between epithelial cells and the surrounding BM/ECM. However, it may also be possible that loss of FBLN2 directly reduces ITGβ1 expression, which in turn leads to a change in COLIV network integrity. Such an impairment of BM integrity upon loss of ITGβ1 has previously been shown in models of lung and kidney morphogenesis^37^ and in epidermal development^38^, while loss of basolateral staining of ITGα2/β1 has been linked to the absence of COLIV expression in breast carcinoma^39^. Also consistent with the hypothesis that FBLN2 provides integrity of the BM, thereby enabling the BM to restrain uncontrolled epithelial outgrowth, is the excessive branching phenotype induced by fibroblasts as seen in our *in vitro* model. However, this branch pattern did neither resemble the controlled *in vitro* branch pattern of EpH4 cells seen in response to growth factors^40^ nor of mammary organoids^18^, where epithelial branching emerges as multicellular buds.

A continuous mammary BM containing COLIV, laminins 111, 332, 511, and 521^5,14,41^ is associated with normal mammary ducts and stable stroma^42^. The results from our study are consistent with those findings, as FBLN2 expression was mainly found around normal mammary ducts and in the interlobular stroma surrounded by a coherent fibrous collagen network (Fig. 6). FBLN2 was neither expressed in the intralobular stroma of normal tissue nor in invasive tissues, with both of them showing a loose fibrous collagen network. Interestingly, at the boundary between morphologically normal mammary duct, ductal carcinoma *in situ*, and invasive area, FLBN2 was expressed in the region of normal duct but not at the invasive cancerous region where BM was absent. This is consistent with data from a Matrigel-embedded system, in which FBLN2 was able to reduce the invasion of breast cancer cell lines (MDA-MB-231 and BT-20)^17^.

The FBLN2 expression pattern in the normal adult human breast around tumour tissue differs strongly from that in the adult mouse where FBLN2 was only detected at times of BM remodelling during very early pregnancy. We have not evaluated human pubertal tissue to assess whether FBLN2 expression is found more strongly in areas of epithelial growth with new BM formation as was seen in the mouse. The reason for this difference in adult tissue expression is not clear, though it may either indicate an anatomical or structural difference in that FBLN2 when incorporated into the ductal matrix of the human breast tissue is still accessible and therefore detectable, while inaccessible and therefore not readily detectable around mouse mammary ducts, or that FBLN2 in the human breast is not only present during the new formation and remodelling of the ducts, but may have functions during duct maintenance in the breast.

Studies have suggested FBLN2 contributes to metastasis and invasion in lung adenocarcinoma^22^, in pancreatic cancer cell lines^43^ and in transformed keratinocytes^44^. However, loss of FBLN2 protein expression has been reported in breast carcinoma^17,45^, and FBLN2 methylation has been demonstrated in five breast cancer cell lines^46^. The absence of FBLN2 in cancerous regions is consistent with our hypothesis that FBLN2 negatively controls invasive growth by interacting with and stabilizing the BM in areas of normal ductal stroma. This was further supported by the lack of FBLN2 expression in the stromal regions of tumours where the metastatic stromal markers TN-C, POSTN, and GAGs^26,47,48^ were all expressed, as well as FBLN2′s presence around normal ducts where these metastatic markers were absent. Such an invasive growth controlling role of FBLN2 would further be explained by the improved DMFS rate for breast cancer patients with LN negative or intermediate to low grade breast cancer, where the level of progression from DCIS to invasive growth could be the distinguishing factor. In contrast, when high grade patient groups were assessed, increased risk of distant metastatic spread in patients was indeed associated with higher expression levels of *Fbln2* mRNA, with a similar trend for LN positive patients. This could indicate that in these groups, in which initial invasion through the BM has already occurred, FBLN2 expression in the tumour cells could be advantageous for their growth during the later stages of progression, or potentially at the final step of micrometastasis formation when the tumour cells home into the tissue of the distant organ to form another malignant tumour. A similar mechanism has been suggested in lung adenocarcinoma, where FBLN2 might be required for the formation of the new ECM around tumour cells possibly originated from tumour-associated fibroblasts^22^. Furthermore, a potential dual role of Fbln2 has been reviewed previously^49^, showing that while *Fbln2* was one of 64 up-regulated RNAs included in a solid tumour-derived metastasis signature^50^, other studies have also described it as a tumour suppressor in several different cancers, a general pattern typical of fibulins^49^. Whether this is due to its role as a BM stabilising protein or due to other factors regulated by FBLN2 is unknown. One such potential candidate is TGFβ, which has been shown to have tumour suppressive activity in early stages of cancer development, while enhancing invasion and metastasis formation in more advanced cancers^51,52^. FBLN2 enhances TGFβ activity^53,54^ and can also compete with latent TGFβ-binding protein for binding to fibrillin-1^55^, thereby regulating TGFβ deposition within the ECM and bioavailability. However, unpublished data from our laboratory have also shown that TGFβ itself can activate FBLN2 expression in EpH4 cells and primary mouse fibroblasts, so that it may be difficult to distinguish between cause and effect. In future studies, it could therefore be important to examine Fbln2′s role in the survival of tumour cells under conditions experienced during metastasis, as well as FBLN2 expression in both the metastasis itself and in the matched primary tumour.

## Materials and Methods

### Cell Culture

EpH4 cells expressing FBLN2 were passaged in 75 cm^2^ tissue culture filter flasks at 37 °C in a humidified atmosphere with 5% CO_2_. Cells were grown in DMEM (Thermo Fisher Inc., Surrey, UK)/10% FBS media (Thermo Fisher) supplied with 2 mM L-glutamine (Thermo Fisher), 100 U/ml penicillin (Thermo Fisher) and 100 µg/ml streptomycin (Thermo Fisher). Mammary fibroblasts were isolated as described previously^56^. Briefly: mammary glands were cut into very small pieces using sterile scalpels and incubated with collagenase A solution (Sigma Aldrich Company Ltd., Dorset, UK) at 2.5 mg/ml and 0.2% trypsin (Thermo Fisher) at 37 °C for 30 min with mild agitation (130 rpm). The tissue homogenate was spun down at 1500 rpm for 10 min, the pellet was resuspended in DMEM/F12 medium, treated with DNase I (Sigma) at 2 U/ml for 10 min, and the DNase was removed by spinning the cells down at 1500 rpm for 10 min. Differential centrifugation was performed 5 times to separate epithelial cells from the stromal compartment. Finally, the stromal compartment was re-suspended in DMEM/F12 medium (Thermo Fisher) with 10% serum, and incubated at 37 °C in a humidified atmosphere with 5% CO_2_.

### Production of lentivirus and transduction of EpH4 cells

For virus production, HEK-293 cells (70–80% confluency) were transfected using X-tremeGENE 9 (Roche Diagnostics Ltd., Burgess Hill, UK) according to manufacturer’s manual with 3 μg pCMV-dR 8.91 (packaging vector, Thermo Fisher) + 0.7 μg pCMV-VSV-G (envelope vector, Thermo Fisher) + 3 μg Lentiviral shRNA vectors (three pLKO1-Puro vectors targeting all *Fbln2* variants of the mouse under control of the human U6 promotor (Clones: TRCN0000109479, TRCN0000109478 and TRCN0000109476 (Thermo Fisher), or a scr ctrl shRNA pLKO1-Puro control vector (Thermo Fisher)). These were mixed in 100 µl of Opti-MEM (Thermo Fisher), 6 μl X-tremeGENE 9 was added, and the transfection solution applied to the cells. After overnight incubation, the medium was replaced with fresh DMEM/10%FBS medium, and incubated for another 24 hrs, after which the virus-containing medium was collected and filtered using syringe-driven filters (Millipore Ltd., Livingstone, UK). Filtered virus was added to EpH4 cells in a 6-well plate, incubated for 4 hrs at 37 °C in a 5% CO_2_ incubator, topped up with DMEM/10% FBS medium + 8 ug/ml polybrene (Sigma) and finally incubated for 24 hrs.

The medium was replaced with fresh medium containing 3 µg/ml puromycin (Sigma) for selection of transduced cells. Cells were routinely passaged with puromycin-medium to maintain the selection.

### Matrigel-embedding and epithelial growth in 3D system

Growth factor-reduced Matrigel™ matrix (Becton Dickinson (BD) UK Ltd., Oxford, UK) was thawed on ice overnight. 100 μl of Matrigel was added to each well of an eight-well chamber slide (Nunc, Sigma) to cover the surface of each chamber, and incubated at 37 °C for 30 min to solidify. 10,000 transduced EpH4 cells per chamber were re-suspended in DMEM-F12/serum free medium mixed with 5% Matrigel and plated on the Matrigel layer in each chamber. Cells were grown in serum free DMEM-F12 medium overnight after which the medium was replaced every other day with DMEM-F12/5% FBS for 7–8 days before microscopic analysis. For co-culture, fibroblasts and EpH4 cells were mixed in a 1:1 ratio and plated as above. For experimental end point quantification, the number of structures on low magnification was counted in two representative images for each condition; the mean number from biological replicates was then calculated.

### Flow cytometry

Fbln2 KD and scr cells were trypsinised, washed with DPBS and re-suspended in DPBS at 1 × 10^6^ cells/ml. 100,000 cells were counted per experiment. Scr cells were used as the reference to assign cell size (Forward scatter, FSC) and granularity (side scatter, SSC) parameters. With the exclusion of dead cells, debris and clumped cells, each line was analysed for these parameters using BD FACSARIA II (BD) provided with FACSDiva Version 6.1.3 software.

### Immunocytochemistry

Cells were fixed in 4% PFA (Sigma) for 20 min at RT followed by 1 min incubation in ice-cold methanol (Sigma). Cells were washed three times with PBS (Thermo Fisher), permeabilised with PBS + 0.5% Triton-100 (Sigma) for 10 min at 4 °C, and then rinsed with 100 mM glycine (Sigma) in PBS three times for 10 min each to reduce auto-fluorescence.

Cells were blocked with blocking buffer (PBS+ 10% FBS, 7.7 mM NaN3 (Sigma), 0.1% BSA (Thermo Fisher), 0.2%Triton (Sigma) x-100, 0.05% Tween-20 (Sigma)) with rocking for 1 hr at RT and then incubated with the primary antibody (FBLN2 at 1:10,000 (kind gift from M-L. Chu^7^), ITGβ1 (BD, 550531) at 1:100, ITGα3 (kind gift from C.M. DiPersio^19^) at 1:400 and COLIV (Abcam, Cambridge Science Park, Cambridge, UK, ab6586) at 1:500) diluted in blocking buffer.

Cells were washed three times with blocking buffer on a rocking platform, and incubated with the appropriate Alexafluor 488- or 594-conjugated secondary antibody (Thermo Fisher) diluted in blocking buffer in the dark for 1 hr at RT. Cells were washed with PBS three times with rocking and finally mounted using Prolong Gold containing DAPI (Thermo Fisher) to counterstain nuclei. 3D spheroids were visualised with a LSM 780 confocal microscope (Carl Zeiss Ltd., Cambridge, UK) using a Z-stacking module, and 2D stained cells were visualised with an Olympus IX51 inverted fluorescence microscope (Olympus Ltd., Surrey, UK) using CellP Imaging software (Olympus).

### RNA Isolation and Quantitative RT-PCR

RNA was extracted using Direct-zol™ RNA MiniPrep (Zymo Research Corporation, Irvine, USA) as per manufacturer’s instructions. The RNA was reconstituted in RNase-free water and quantified using the Nanodrop ND-1000 Spectrophotometer (Thermo Fisher). cDNA was produced using 200–500 ng RNA with Superscript II (Thermo Fisher) and random primers according to manufacturer’s instructions. PCR reactions were carried out using a LightCycler® 480 Instrument (Roche) with the appropriate Lightcycler probes under standard conditions. Each reaction mixture contained 1 μl (0.25 μM) of probe, 1 μl of primer mixture (7.2 μM of both forward and reverse primers), 10 μl of 2x LightCycler® 480 TaqMan Master Mix (Roche), 5 μl of diluted cDNA and dH_2_O to a final reaction volume of 20 μl. The following primers (Sigma) were used: FBLN2: Fwd 5′- tgttgttggggacacagcta −3′, Rev 5′- ccatcaaacactcgtcttggt −3′, Probe (#22), Beta actin: Fwd 5′- aaggccaaccgtgaaaagat −3′, Rev 5′- gtggtacgaccagaggcatac −3′, probe (#56). Relative RNA expression was calculated by the 2^−ΔΔCt^ method.

### Protein isolation and western blotting

Cells were collected and lysed in ice-cold RIPA buffer (25 mM TRIS (Sigma) pH 8, 150 mM NaCl (Sigma), 1% NP-40 (Sigma), 1% sodium deoxycholate (Sigma), 0.1% SDS (Sigma), with protease inhibitor cocktail (Roche) and phosphatase inhibitors (sigma) in dH_2_O. The suspension was pipetted up and down through a 1 ml syringe to break up cells and chromatin, and kept on ice for 5 min. The cell lysate was centrifuged at 4 °C for 20 min at 21,000 g and the soluble protein in the supernatant was quantified using a Pierce™ BCA Protein Assay Kit as per manufacturer’s instructions (Thermo Fisher). 50 μg of denatured (in 1xLDS-loading buffer with 0.1 M DTT) protein per sample was separated on a Novex NuPage™ Electrophoresis (Thermo Fisher) system using 4–12% Bis-Tris-HCl buffered gels in the presence of 1xNuPage MES SDS running buffer (Thermo Fisher). Proteins were transferred onto Whatman® Protran® Nitrocellulose Transfer Membrane (0.2 μm) (GE Healthcare Bio-Sciences, Pitsburgh, PA, USA) using a Novex XCell II™ Blot module (Thermo Fisher). Successful transfer was confirmed by PonceauS (Sigma) staining. The blot was then incubated for 40 min at RT in blocking solution (3–5% dried skimmed milk powder in wash buffer (1X PBS/0.1% Tween-20)), followed by incubation for 2 hrs at RT with the appropriate primary antibody (goat anti-actin at 1:200 (Santa Cruz Biotechnology INC., Santa Cruz, USA, Sc-1615), rabbit anti-FBLN2 at 1:10,000, rabbit anti-ITGβ1 at 1:500 and rabbit anti-ITGα3 at 1:1000. After three 10 min washes with wash buffer, the blot was incubated for 1 hr at RT with horseradish peroxidase (HRP)-labelled secondary antibody (Thermo Fisher) in blocking buffer, and finally washed three times with wash buffer for 10 min.

A chemi-luminescence reaction was carried out using the ECL Western blotting detection reagent kit (Thermo Fisher) as per manufacturer’s instructions. The signal was detected using an Intelligent Dark Box LAS-3000 (Fujifilm UK Ltd., Sheffield, UK) equipped with a cooled CCD camera. Images were acquired and analysed using LAS-3000 v.2.2 software (Fujifilm).

Filters were cut into the appropriate size ranges (at ~60 kDa) to allow for multiple antibody incubation of the same samples (FBLN2/actin; ITGβ1/actin). Where this was not possible, filters were stripped under mild conditions (15 min in 0.1% SDS/0.1% Tween-20 (pH2)) and complete removal of antibodies was tested before re-incubation with another primary antibody (FBLN2/ITGα3).

### Human patient data

65 breast cancer patients were recruited from breast clinics at Cairo University, Kasr Alainy Hospital upon signed informed consent from all participants and with the acceptance of Cairo University ethical committee (**IRB: N-8–2017**). All research was performed in accordance with relevant guidelines and regulations. Patients’ ages ranged from 32 to 72 years old, with mean age 53 ± 12. Clinical and pathological features of patients’ cohort including tumour grade, lymph node (LN) involvement, lymph-vascular invasion were established and the expression of hormone and growth factor receptors (ER, PR and HER-2) all assessed by IHC (Supplementary Table 1).

Pathological grade of tumour and margins of surgical excision were estimated using haematoxylin & eosin archival stained sections. Normal breast ducts were identified by an intact basement membrane, double epithelial and myoepithelial cell lining with regular nuclei and fine chromatin. Areas of DCIS were defined by regular rounded contours, intact basement membrane with peripheral myoepithelial layer, and filled with multiple layers of malignant epithelial cells having large nuclei coarse chromatin and occasional nucleoli. Invasive carcinoma was defined as having formed small or large groups of malignant epithelial cells, with some forming tubules with irregular contours, with neither myoepithelial layer nor basement membrane present.

### Fibrous collagen and GAG staining

5 μm sections routinely cut from formalin fixed, paraffin wax-embedded (FFPE) tissue sections were deparaffinised in xylene (Sigma) for 10 min, re-hydrated in decreasing concentrations of ethanol (Sigma) and finally soaked in tap water. For collagen I and III staining, slides were incubated in Picrosirus red stain (Abcam) for 1 hr and then washed in 0.5% acetic acid (Sigma) for stain differentiation. For GAG staining, slides were incubated in Alcian blue stain (Sigma) for 20 min^57^ and counterstained with eosin (Dako UK Ltd., Eli, UK). Slides were then dehydrated in increasing concentrations of ethanol, cleared in two changes of xylene and finally mounted with DPX mounting medium (Sigma).

### Immunohistochemistry of human breast sections

Antigen retrieval was performed on 5 μm FFPE human breast tissue sections using 1 mM EDTA (Sigma) buffer (pH 8) under high pressure and all other incubations were performed at RT using a humidified chamber. Sections were blocked with pre-diluted 2.5% goat serum (Thermo) for 20 min and incubated with primary antibody for 30 min. All antibodies were diluted to their final concentrations using Antibody Diluent (Dako) (FBLN2 1:10,000; COLIV 1:400 (Abcam, ab6586); TN-C 1:200 (Abcam, ab108930); POSTN 1:150 (Santa Cruz, Sc-49480); Vimentin (Vim) 1:100 (Abcam, ab8978); Smooth muscle actin (SMA) 1:200 (Thermo Fisher, MS-113P). Sections were washed thrice and then incubated for 30 min with HRP-conjugated goat polyclonal anti-rabbit/anti-mouse (Dako) for FBLN2, COLIV, SMA, Vim and TN-C, and horse anti-goat for POSTN (HRP labelled) secondary antibody diluted 1:500 (Vector Laboratories Ltd.; Peterborough, UK), prior to staining with DAB + Chromogen (Dako) for 4 min. Stained tissue sections were counterstained with haematoxylin (Dako), dehydrated through increasing concentrations of ethanol and xylene before mounting with cover slips using DPX mounting medium (Sigma). Negative controls (lacking primary antibody) were used in each staining run. All 65 breast tumours were assessed for FBLN2 expression, while COLIV, POSTN, SMA, Vim and TN-C were each assessed in 10 randomly chosen consecutive sections. Scoring of FBLN2 expression was performed based on visual assessment of staining intensity and the number of stained structures, and was given values from 0 (negative) to 3 (strong).

### Statistical analysis

FBLN2 expression in normal, DCIS and invasive regions in the 65 patients’ sections was analysed for associations with hormone receptors (ER, PR) and growth factor receptor (HER2) expression as well as histological grade. A Pearson’s test was used to assess the association between the score number of each region and the other parameters. All tests were two sided. P-values < 0.01 were considered statistically significant.

## Electronic supplementary material

Supplementary Figures and Tables with legends
