# Supplementary material for: Fibulin-2 is required for basement membrane integrity of mammary epithelium
Source: Sci Rep. 2018 Sep 20;8:14139. doi: 10.1038/s41598-018-32507-x (PMC6148073; doi:10.1038/s41598-018-32507-x)
Supplement: Supplementary file 1 — Supplementary Figures and Tables with legends [file 41598_2018_32507_MOESM1_ESM.pdf]

Supplementary Figures and Tables with Legends

Fibulin-2 is required for basement membrane integrity of mammary epithelium

Ayman M. Ibrahim[1,2*], Salwa Sabet[1], Akmal A. El-Ghor[1], Nora Kamel[3], Shady E. Anis[4], Joanna S. Morris[5], Torsten Stein[2*]

[1] Zoology Department, Faculty of Science, Cairo University, Giza 12613, Egypt

[2]Institute of Cancer Sciences, College of MVLS, University of Glasgow, Glasgow, G12 8QQ, UK

[3]Department of Pathology, National Research Center, Cairo 12622, Egypt

[4]Department of Pathology, Faculty of Medicine, Cairo University, Cairo 11562, Egypt

[5]School of Veterinary Medicine, University of Glasgow, Bearsden Road, Glasgow G61 1QH, UK

Supplementary Figure 1

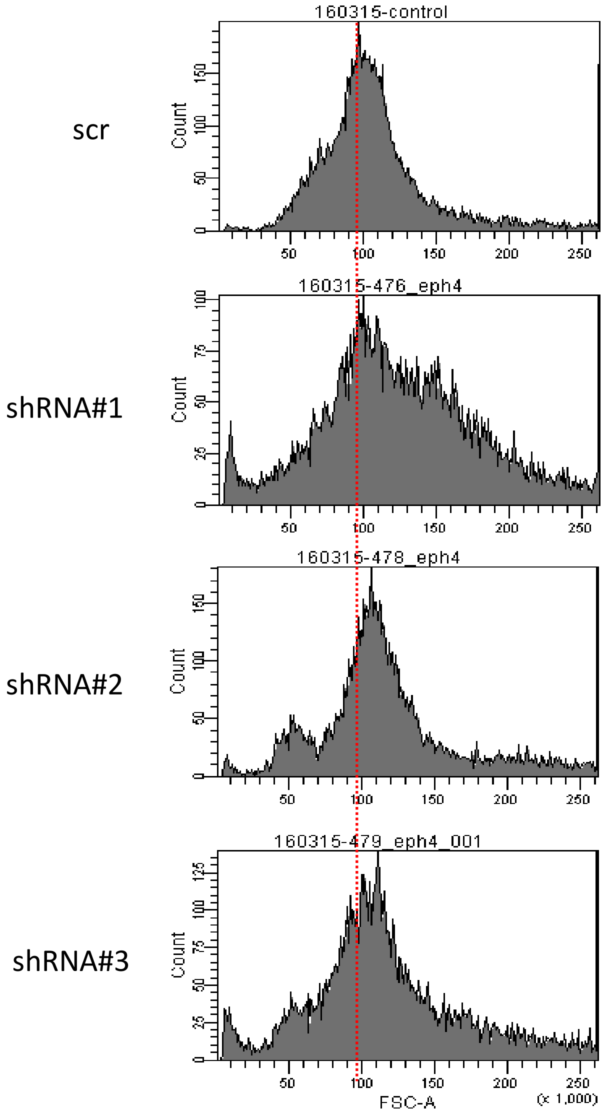

Supplementary Figure 2

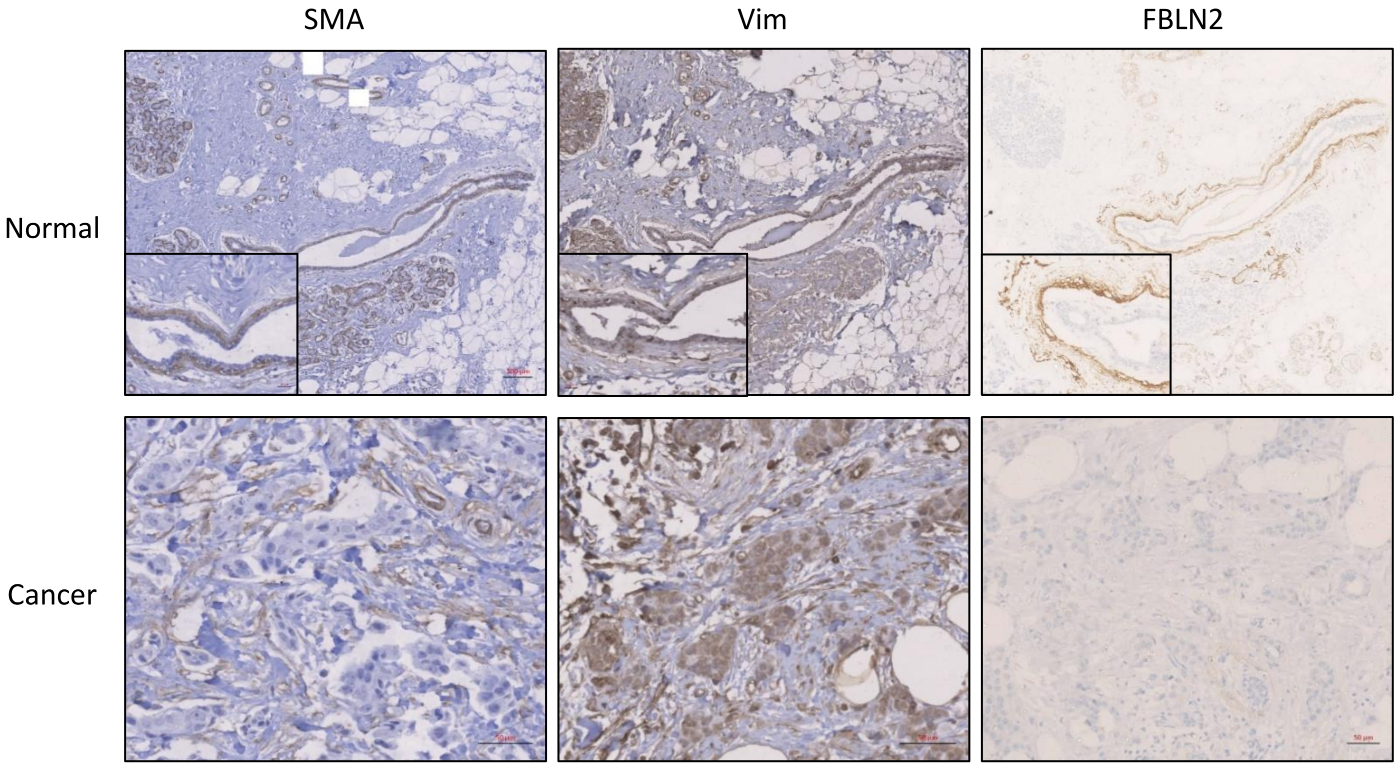

Supplementary Figure 3

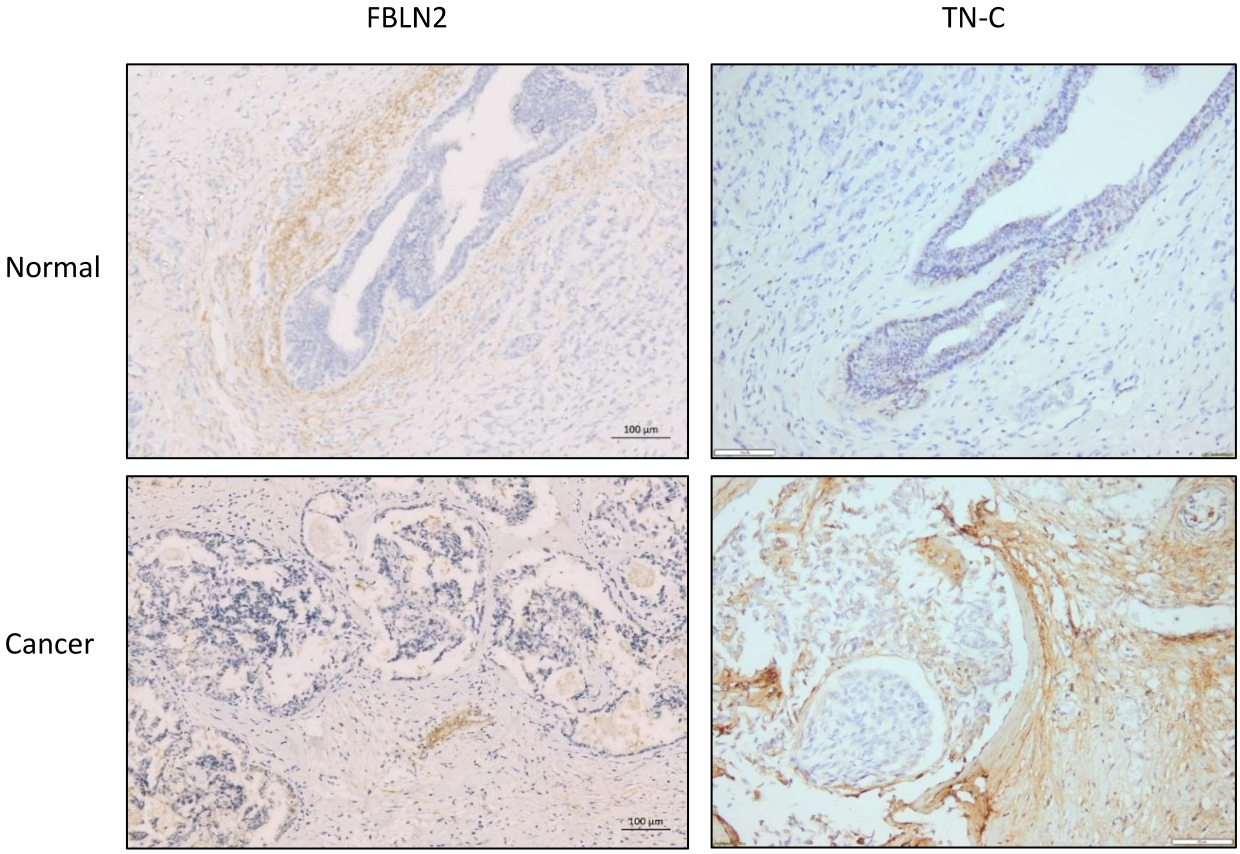

Supplementary Figure 4

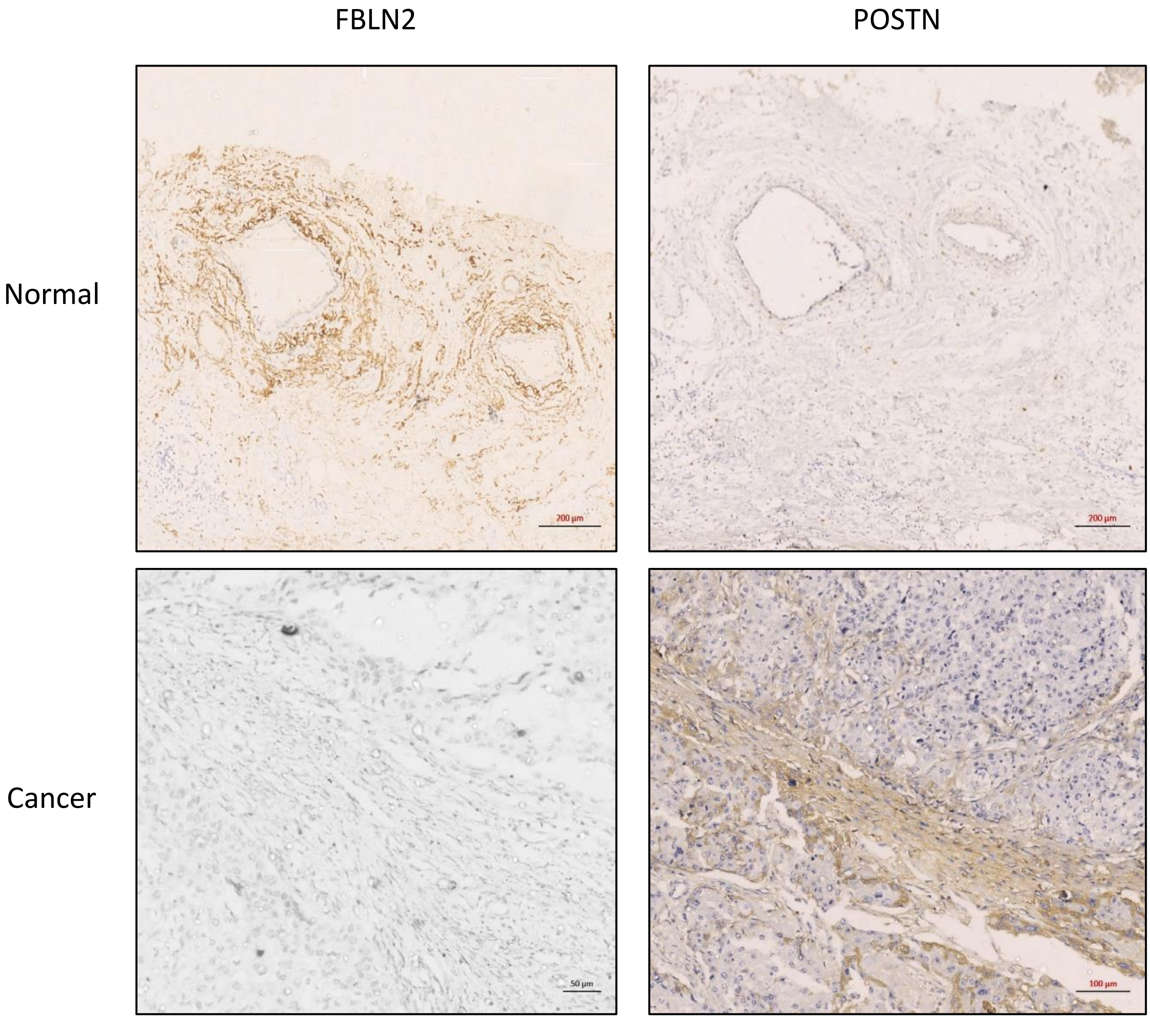

Supplementary Figure 5

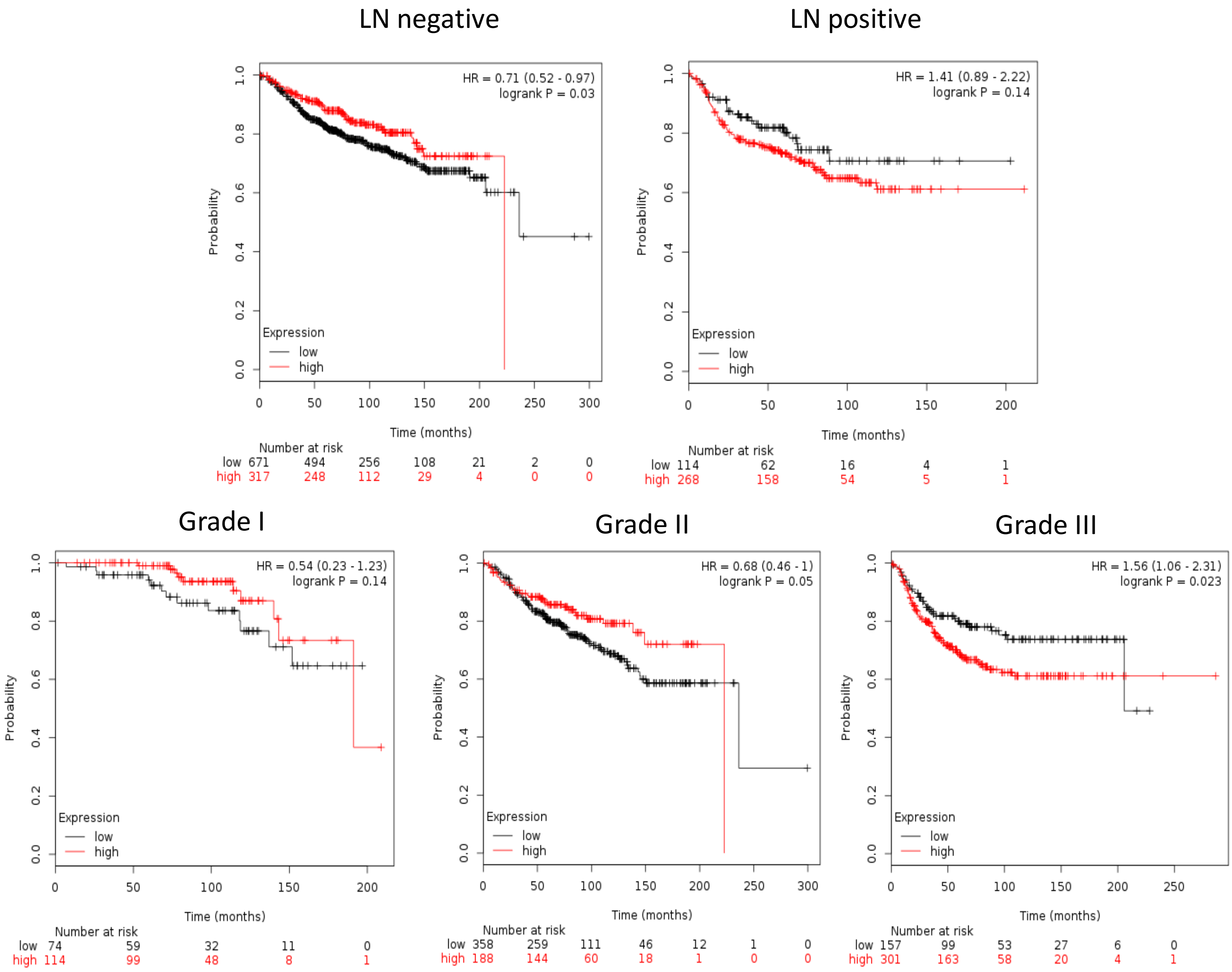

Supplementary Figure 6

Chemiluminescence mode

Chemiluminescence mode

Colorimetric mode

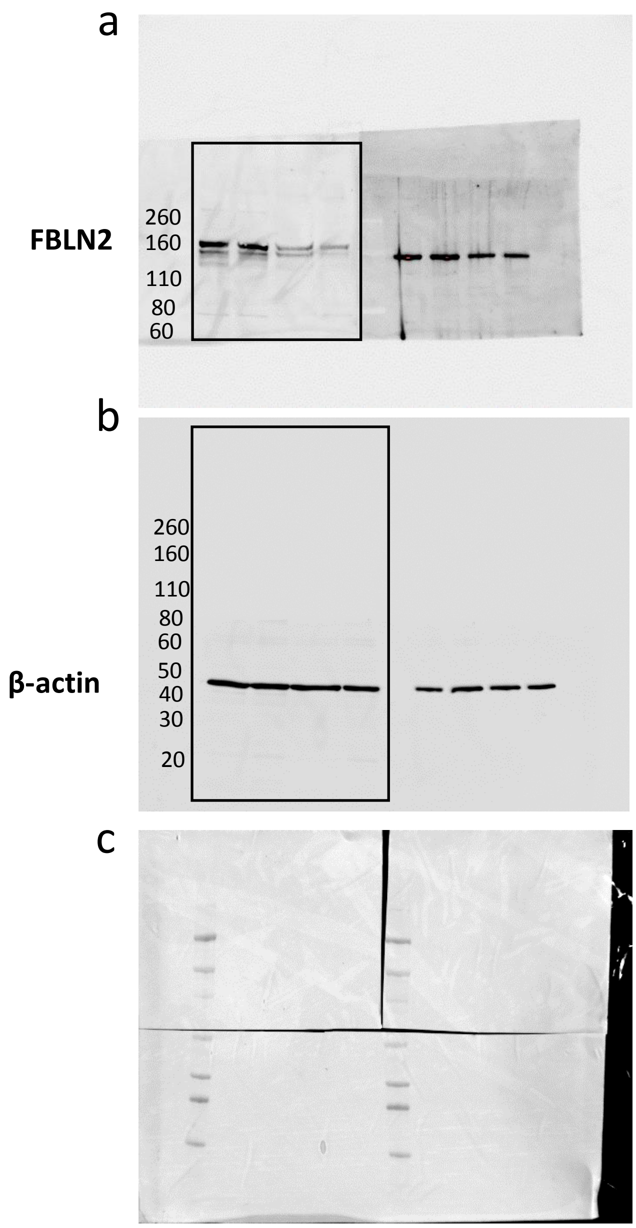

Supplementary Figure 7

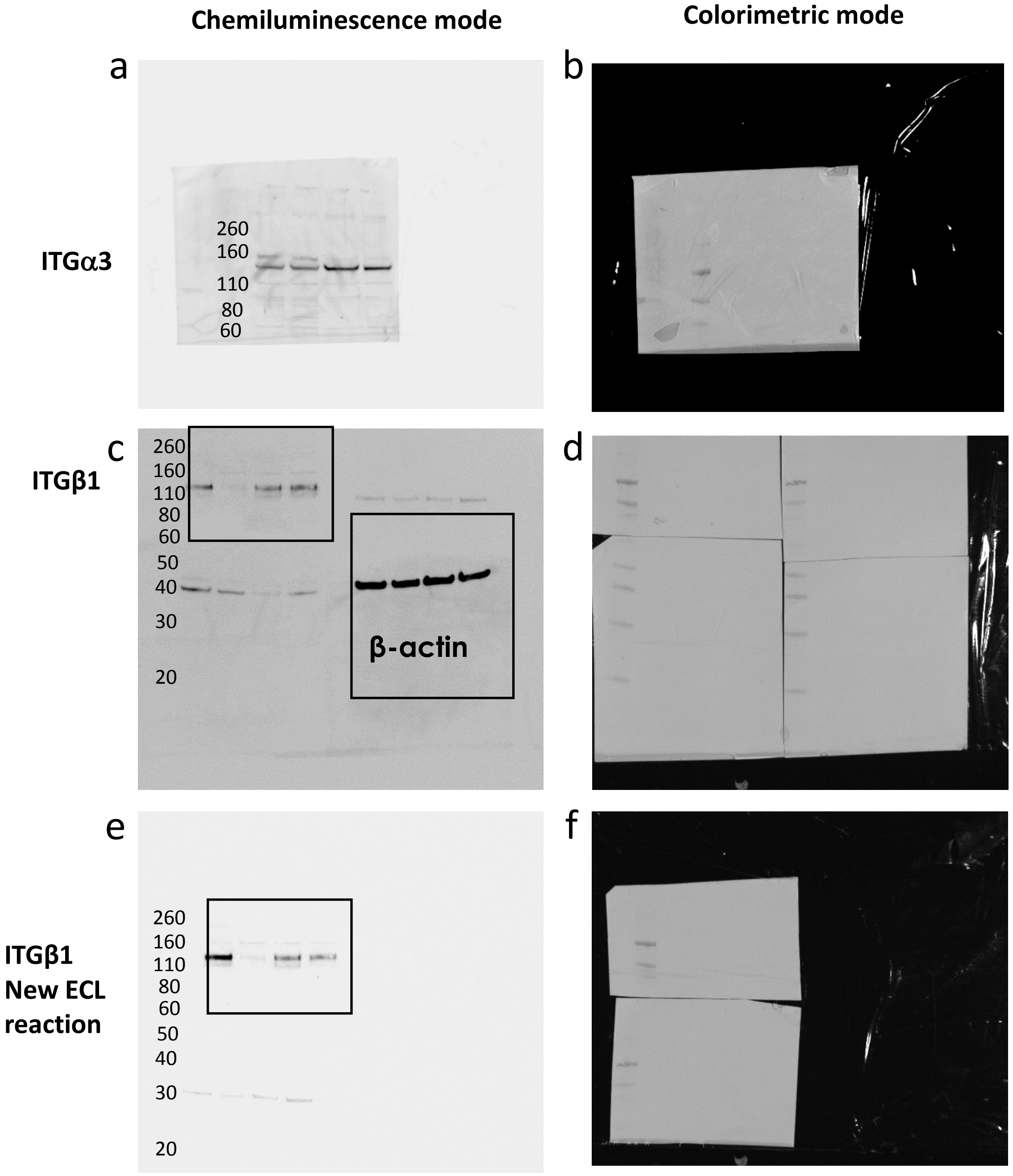

Supplementary Table 1

Parameter	Number of patients (n =65) (%)
**Age (yrs)**	
Range	32-72
Mean	53±11.98
**Number of positive Lymph nodes**	
<4	44 (68%)
≥4	21 (32%)
**Tumour Grade**	
Grade II	59 (91%)
Grade III	6 (8%)
**ER status**	
Positive	44 (70%)
Negative	20 (30%)
NA	1
**PR status**	
Positive	37 (59%)
Negative	27 (41%)
NA	1
**HER-2 status**	
Positive	11(18%)
Negative	53(82%)
NA	1

Supplementary Table 2

Clinical parameter		FBLN2 SCORE																	
		Normal						DCIS						Invasive					
		total	0	1	2	3	Pearson correlation/ P value	total	0	1	2	3	Pearson correlation/ P value	total	0	1	2	3	Pearson correlation/ P value
ER	ER positive	42	0	0	14	28	0.172/0.186	17	13	4	0	0	-0.005 / 0.971	42	31	11	0	0	-0.016 / 0.901
	ER negative	19	2	0	5	12		39	30	9	0	0		18	13	5	0	0	
	NA				1										1				
PR	PR Positive	36	0	0	12	24	0.139/0.287	34	26	8	0	0	.009/ 0.946	35	26	9	0	0	-.025/0.847
	PR negative	25	2	0	7	16		22	17	5	0	0		25	18	7	0	0	
	NA				1										1				
HER2	HER2 Positive	10	2	0	1	7	-0.194 /0.134	9	8	1	0	0	-0.130 / 0.343	9	6	3	0	0	0.063/0.631
	HER2 negative	51	0	0	18	33		46	34	12	0	0		51	38	13	0	0	
	NA				1				1						1				
Grade	Grade II	56	1	0	16	39	-0.439 / <0.001*	50	38	12	0	0	-0.027 / 0.844	54	40	14	0	0	0.050/0.703
	Grade III	5	1	0	4	0		5	4	1	0	0		6	4	2	0	0	
	NA					1			1						1				

Supplementary Figure Legends

Supplementary Fig. 1 Flow Cytometric analysis of cell sizes of ctrl and Fbln2 KD cells

Flow cytometry profiles based on forward scatter (FCS) of the stably transduced EpH4 cells (shRNA #1-3) and cells transduced with a scrambled control vector (scr)

Supplementary Fig. 2 FBLN2 expression relative to SMA and Vim staining in areas of morphologically normal tissue and invasive cancer

Immunohistochemical analysis of FBLN2, SMA and Vim expression in normal (top row) and cancerous breast tissues with (bottom row) shows that FBLN2 localises to the stroma surrounding morphologically normal ducts, but is not detected within areas of invasive cancer. Bars represent 50 µm

Supplementary Fig. 3 Immunohistochemical assessment of FBLN2 expression in relation to tenascin C (TN-C)

Immunohistochemical analysis of TN-C expression in comparison to FBLN2 in stroma surrounding morphologically normal ductal epithelium (top), and in areas of tumour invasion (bottom) within the same section. The two proteins show an inverse expression pattern in normal and cancerous tissue. Bars represent 100 µm

Supplementary Fig. 4 Immunohistochemical assessment of FBLN2 expression in relation to periostin (POSTN)

Immunohistochemical analysis of POSTN expression in comparison to FBLN2 in stroma surrounding morphologically normal ductal epithelium (top), and in areas of tumour invasion (bottom) within the same section. The two proteins show an inverse expression pattern in normal and cancerous tissue. Bars represent 100 µm

Supplementary Fig. 5 High levels of Fbln2 mRNA are associated with improved distant metastasis free survival in lymph node negative and intermediate grade breast cancer patients

Kaplan-Meier plots for distant metastasis-free survival showing the stratification of breast cancer patient subgroups LN negative (n=988), LN positive (n=382), grade I (n=188), II

(n=546) and III (n=458)) with high (red) and low (black) levels of *Fbln2* RNA expression. The numbers below each plot are the numbers of individuals at risk in each group.

Supplementary Fig. 6 Full western blots for FBLN2 with original markings

Full scanned western blot shown in **Figure 1a**. The blot was cut at 60KDa; the upper section was probed for FBLN2 (a) and the lower section was probed for β-actin (b). The colorimetric image (c) shows where the membrane was originally cut.

Supplementary Fig. 7 Full western blots for ITGβ1 and ITGα3 with original markings

Full scanned western blot shown in **Figure 4b**. (a) The blot used for FBLN2 visualization, was stripped and re-probed for ITGα3. (c) Samples were loaded with the same concentration twice on the same gel to assess 4 different proteins; in the left half, the blot was cut at 80 KDa; the upper section was probed for ITGβ1, and in the right half, the blot was cut at 60 KDa; the lower section was then probed for β-actin. (e) The chemiluminescence reaction was repeated for better ITGβ1 visualization due to overall low signal in the previous blot in (c). The colorimetric images (d, e and f) show where the membranes had originally been cut.

Supplementary Table Legends

Supplementary Table 1: Clinical and pathological characteristics of the assessed patient cohort (age range, histological tumour grade, number of lymph nodes affected, ER-, PR- and HER-2 status as assessed by immunohistochemistry). NA: No available data

Supplementary Table 2: Pearson's correlation assessment of FBLN2 expression by intensity score (0=negative; 1=weak; 2=intermediate; 3=strong) in morphologically normal margins, areas of DCIS, and invasive areas, as well as with hormone receptors (ER, PR), growth factor receptor (HER2) and tumour grade. NA: No available data
